# Enhancing military airway suction devices with a focus on performance and portability

**DOI:** 10.1186/s12873-025-01262-4

**Published:** 2025-07-16

**Authors:** Maria J. Londono, Saketh R. Peri, Rakib Hasan, Connor J. Evans, David Restrepo, Robert A. De Lorenzo, R. Lyle Hood

**Affiliations:** 1 Department of Mechanical, Aerospace, and Industrial Engineering, University of Texas at San Antonio, One UTSA Circle, San Antonio, TX 78249 USA; 2 Department of Emergency Medicine, University of Texas Health Science Center at San Antonio, 7703 Floyd Curl Drive, San Antonio, TX 78229 USA; 3 Department of Biomedical Engineering & Chemical Engineering, University of Texas at San Antonio, One UTSA Circle, San Antonio, TX 78249 USA

**Keywords:** Airway suction, Medical device, Emergency medicine, Military medicine, Pre-hospital

## Abstract

**Background:**

Airway management is critical in combat casualty care, with airway compromise being the second leading cause of preventable battlefield deaths. Suction devices are essential for clearing obstructions during airway management; however, many medics choose not to carry them due to their excessive weight. Current standards for suction devices mention a minimum liquid flow rate of 1.2 L/min and a maximum device weight of 6 kg, but these standards fail to meet the practical needs of military end-users. The team conducted an I-Corps funded end-user assessment study with over 100 participants, in which a minimum flow rate of 1 L/min and a maximum weight of 4.5 kg were indicated as preferred among respondents. This gap between the standards and user preferences results in exclusion of existing devices from military kits due to weight concerns despite meeting performance criteria.

**Methods:**

To address this gap, the Suction Combat Ready Advanced Multifunctional Machine (SCRAMM) was developed with input from U.S. Military clinical stakeholders to emphasize both performance and portability. SCRAMM is designed to handle diverse medical scenarios simultaneously and was characterized against the market leaders Zoll 330 and Impact 326M. Liquid flow rates and device weights were measured and analyzed according to ISO standards and end-user requirements.

**Results:**

Zoll 330 and Impact 326M exceeded the ISO-required liquid flow rate by 145%, with weights of 4.8 kg and 5.1 kg, respectively. Additionally, both devices were heavier than the user-preferred weight limit of 4.5 kg. SCRAMM, with three suction lines for simultaneous diverse medical tasks, exceeded the ISO flow rate by 23%. It remained within the preferred weight range at 3.4 kg, demonstrating greater performance-to-weight balance in consideration of actual user needs.

**Conclusion:**

This study demonstrates the successful development and characterization of SCRAMM. It met ISO flow rate standards and remained under the 4.5 kg weight threshold preferred by end-users—outperforming current market leaders in portability while maintaining effective suction. These results highlight the importance of incorporating a performance-to-weight metric in evaluating portable suction devices. We recommend that future standards balance performance with portability to better suit military and emergency medical needs. Clinical trial number: not applicable.

**Supplementary Information:**

The online version contains supplementary material available at 10.1186/s12873-025-01262-4.

## Introduction

In 2024, Airway management was identified as a critical research priority in combat casualty care by the Defense Health Agency [1]. Notably, airway compromise is the second most common cause of preventable battlefield deaths and is responsible for more than 10% of mortalities [2–5]. However, life-saving interventions for airway management are frequently overlooked, with over 50% of opportunities to secure the airway in battlefield casualties being missed [2, 3, 6–8]. Addressing this challenge is critically dependent on having the appropriate equipment [9, 10]. The consequences of inadequate airway management extend beyond immediate mortality, as prolonged airway compromise can lead to hypoxia, secondary brain injury, and multi-organ failure, further complicating evacuation and increasing the burden on field medical units [11]. In combat settings, where hazardous conditions and delays in evacuation are common, rapid airway clearance is critical for improving survival rates [12].

Suction plays a vital role in airway management by rapidly clearing obstructions and often precedes the placement of an airway securement device when necessary [6, 13–16]. Given their importance, portable suction devices are essential tools in combat medics’ kits [16–18], enabling the rapid removal of obstructions such as blood, vomit, and debris from the airway [12, 19–24]. Suction is integral to at least three of the ten core essential medical capabilities upon which Prolonged Field Care (PFC) is structured: Ventilate and Oxygenate [25], Airway Management [6, 26–28], and Ongoing Nursing Procedures [29, 30], such as oro/nasogastric suctioning [30, 31]. Beyond airway management, suction devices are also crucial for various other medical procedures including oro/nasotracheal suction for intubated patients [32], surgical suction, gastrointestinal abdominal drainage, wound drainage, pleural mediastinal drainage, nasal gastric drainage, and subglottic secretion removal, among other medical procedures [5, 17, 28, 33, 34]. Additionally, chemical, biological, radiological, nuclear, and explosive (CBRNE) threats can create significant suction requirements. This includes airway burns from conventional and nuclear blasts and bronchorrhea from nerve agents and vesicants [35].

Field hospitals, ground and air evacuation vehicles, and individual combat medics are expected, by their respective professional guidelines, to be equipped with suction devices [17, 24]. The commercial portable/luggable suction devices are categorized into manually powered [36] and electrically powered devices [37]. While manually powered devices are lighter and more portable, they often lack the vacuum necessary for critical tasks like oropharyngeal and airway management in emergency situations [28, 38, 39]. Electrically powered devices, on the other hand, provide the suction power required for these lifesaving procedures, but their size and weight make them impractical for use in military mobile hospitals or remote environments [28].

Because only powered suction devices can meet the performance needs for effective airway management, they are the focus of this analysis. However, despite their life-saving capabilities, many combat medics and first responders choose not to carry these devices due to their bulkiness and elevated weight [38–41]. Market analysis and interviews have highlighted an urgent need for suction devices that balance portability and performance, and this trade-off continues to be a major challenge in their design.

To address these challenges, end-user needs were gathered and synthesized into the design of a new device named the Suction Combat Ready Advanced Multifunctional Machine (SCRAMM). This study, in addition to presenting the novel suction device, aims to characterize the novel prototype alongside two established market leaders used by U.S. Military Forces: the Zoll 330 Multifunction Aspirator and the Impact Ultra-lite 326M.

In recognizing the need for lightweight medical equipment - which was a design requirement identified during a National Science Foundation (NSF) I-Corps program with military clinical end-users and related stakeholders conducted by this group [42] - this study proposes an additional metric to evaluate suction devices based on performance relative to weight. This approach ensures the devices are both effective and portable to better meet the needs of military and civilian medical responders. We hypothesize that SCRAMM - designed with direct end-user input - will provide sufficient suction for effective airway clearance while being significantly lighter than existing market-leading suction devices, making it a more portable and practical solution for military and emergency medical applications. The primary aim of this study is to develop and characterize SCRAMM, a novel portable suction device optimized for both performance and portability. The secondary aims are to (1) evaluate SCRAMM’s suction performance and portability compared to the Zoll 330 and Impact 326M, and (2) analyze weight limitations in current suction devices to inform future design considerations. The study aims to bridge gaps in current standards by providing a more accurate characterization of suction device performance in practical scenarios.

## Methods

The goal of this study was to design a novel multifunctional suction device, SCRAMM, based on end-user input gathered through an NSF I-Corps program with military clinical stakeholders [42]. This design aimed to balance performance and portability, while addressing challenges identified in battlefield use. SCRAMM was evaluated alongside the Zoll 330 and the Impact 326M with a focus on liquid flow rates, weight, and the development of a performance-to-weight ratio metric that better aligns with field needs.

**Fig. 1:**
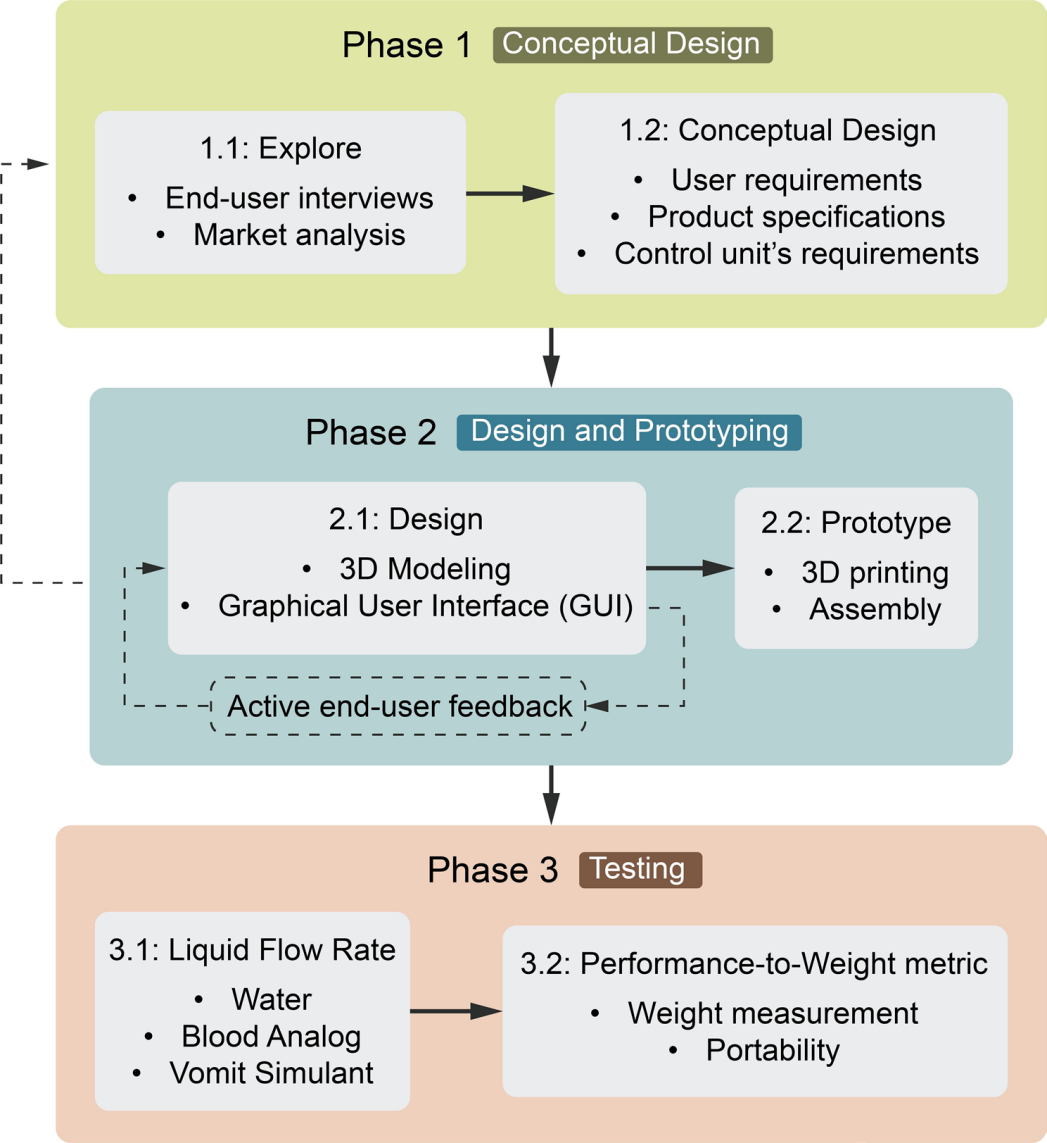
Summary of the study methodology including a conceptual design phase, a design and prototype phase and a testing phase

The methodology outlined a structured approach with three stages: conceptual design, detailed design and prototyping, and testing, as outlined in Fig. 1. In the conceptual design phase, user requirements were gathered through interviews and market analysis to ensure the design would meet real-world needs. The detailed design and prototyping phase focused on three-dimensional (3D) modeling, graphical user interface (GUI) development, and prototype assembly while incorporating continuous feedback from end-users. Finally, the testing phase evaluated the device’s performance via measurement of liquid flow rate using various simulants and performance relative to weight to ensure that the device was both functional and portable.

### Phase 1: Conceptual design

To prioritize the essential characteristics of a portable suction device suitable for prehospital care, the team initially conducted a series of surveys and interviews through the U.S. NSF National I-Corps program. A total of 102 interviews were conducted with military stakeholders including combat medics, emergency medicine physicians, U.S. Special Forces personnel, paramedics, emergency medical technicians, supply chain experts, manufacturing representatives, U.S. Food and Drug Administration (FDA) consultants, and police officers from Texas and the Washington D.C. area [28]. The interview questions were specifically developed for this study as part of the I-Corps program and were not adapted from previously published sources. The full set of questions can be found in Additional File 1. Additionally, an analysis of leading market devices such as the Zoll 330 and the Impact 326M was performed.

The insights gathered from these interactions formed the foundation for the design, which was tailored to meet the identified need for a lightweight device that could still deliver reliable suction performance. Weight was consistently identified as a major limitation for existing devices, with medics emphasizing that current electrically powered suction units were too heavy to be practical for field use. To address this, the team established a maximum weight threshold based on user input, ensuring the device remained within the acceptable carry limits reported by military personnel. This requirement directly influenced the selection of materials and internal components, prioritizing lightweight yet durable alternatives to avoid unnecessary overdesign. In addition, users highlighted the need for compact storage and transportability, leading to the adoption of a form factor that allowed easy integration into existing medical kits. These constraints guided key trade-offs in suction power, battery capacity, and overall device dimensions to improve usability in combat environments.

### Phase 2: Detailed design and prototyping

Based on user feedback and the recognition of the limitations in existing market devices, the SCRAMM device was developed to achieve a balance between performance and portability. The design methodology was based on a user-centered design approach [43]. The process involved creating 3D models to ensure precision in both aesthetic and functional aspects. A key focus of this design was enhancing the multifunctionality of the device, enabling it to perform parallel operations across three independent suction lines, each tailored to different medical scenarios requiring specific suction pressures.

These models were refined through nine major iterations, with significant modifications informed by user's direct feedback, such as the transition from non-conventional shapes to a book-style form factor. Throughout the design iterations, it became evident that performance and weight had to be jointly optimized. By maintaining a focus on portability while ensuring high suction power, the design aimed to meet the operational standards for field use.

The device’s GUI was also developed with integrated alarm signals based on competitor analysis, user feedback, and compliance with International Organization for Standardization (ISO) 10079–1 guidelines [44] to ensure that the final design met both user needs and regulatory requirements. The SCRAMM prototype was then constructed using a combination of commercially available components and in-house fabricated parts produced with stereolithography (SLA) 3D printing technology.

### Phase 3: Testing

The devices selected for this study were chosen based on their usage within the U.S. Military Forces and their relevance to battlefield medical scenarios. The devices tested included SCRAMM - a novel design - alongside the current market leaders: the Zoll 330 and the Impact 326M.

The performance of the devices was evaluated through liquid flow rate tests using fluids of varying viscosities. Testing protocols adhered to ISO 10079–1 guidelines and previous studies [44–46], with five trials conducted for each device (*n = 5*). A power analysis determined the number of trials, allowing for a 5% margin of error on a flow rate of 1.5 L/min, with a 95% confidence level.

The performance tests aimed to assess the devices’ liquid flow rates using water and an ISO-specified blood analog and vomit simulant mix to simulate real-world medical scenarios [44]. These simulants were selected to represent the range of fluids encountered in battlefield airway management. Water served as a baseline, while the blood analog and vomit simulant were chosen to mimic the viscosity and composition of bodily fluids that commonly obstruct airways in combat casualties, such as hemorrhagic secretions and regurgitated contents. This ensured that the suction performance was evaluated under conditions that closely resemble those faced in field trauma care. It is important to note that there are no universally accepted standards for battlefield fluid simulants. Therefore, members of the research team have developed various techniques to approximate real-world scenarios while ensuring reproducibility in testing conditions. As per ISO 10079–1 standard, a minimum liquid flow rate of 1.2 L/min for viscous solutions such as vomit simulant is required [44]. These tests were conducted to verify compliance with this specification. The liquid flow rate test setup was designed in accordance with established protocols from previous suction device performance evaluations detailed in [45]. In addition to flow rate, the weight of each device was measured using a calibrated scale and compared against the thresholds derived from end-user interviews and medical device standards.

To improve reproducibility, the testing setup was calibrated before each trial using standard calibration test weights. Between trials, devices were flushed with water and inspected for blockages to prevent residual fluid buildup from affecting subsequent measurements. Each trial followed a standardized protocol, ensuring consistent fluid volume and suction duration across all tests.

The resulting data were visualized to show the performance-to-weight ratio, illustrating the operational importance of balancing these factors in field conditions.

## Results

### Phase 1: Conceptual design

Interviews with end-users emphasized the critical importance of both weight and flow rate for the device. The excessive weight of commercially available suction devices was cited as the primary reason for their limited use in combat scenarios. While ISO 10079–1 standards set a maximum allowable weight of 6 kg for electrically powered suction devices and a required flow rate of 1.2 L/min for viscous solutions [44], military end-users suggested different requirements for field conditions. Specifically, they indicated that a device weighing no more than 4.5 kg would be practical in military in-field hospital settings, and a flow rate of 1 L/min would be sufficient to clear airway obstructions. Additionally, users expressed a need for multifunctionality, with the device capable of addressing diverse medical scenarios. These are some of the key requirements and specifications; the complete list can be found in Additional File 2. Additionally, feedback from military end-users revealed the importance of optimizing the trade-off between weight and suction power, and this shaped the development focus of SCRAMM to ensure that the device would be both functional and portable in field conditions.

### Phase 2: Detailed design and prototyping

**Fig. 2:**
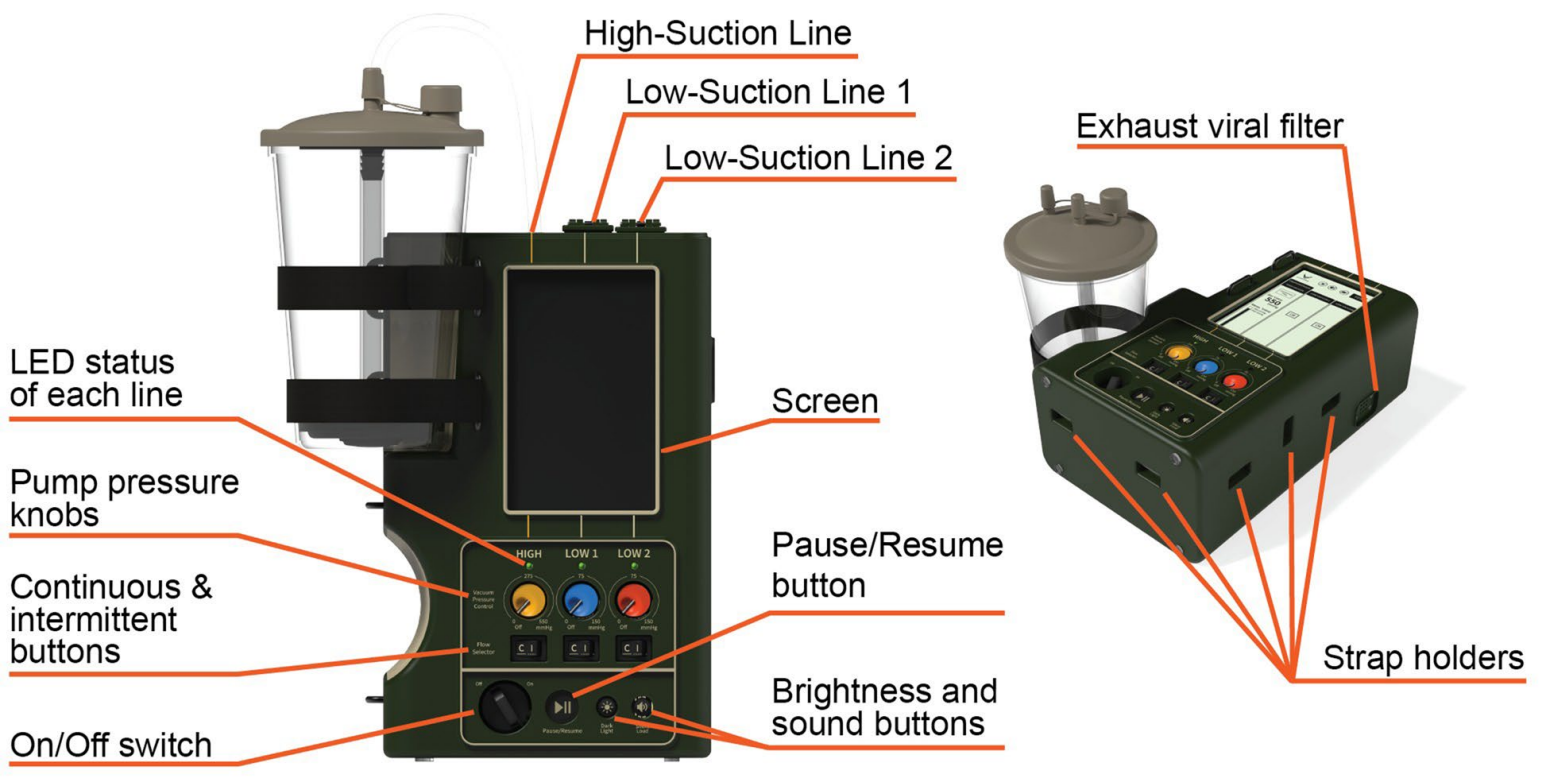
SCRAMM’s final version with highlighted key features, including the control layout, LED indicators, suction line pressures, and color-coded functions (yellow, blue, red) to simplify operation in high-stress environments

The development of the SCRAMM device followed an iterative process, with each design phase incorporating significant changes based on user assessment and engineering insights. Initially compact, the design evolved into a more rectangular form with an integrated handle and multiple canisters to enhance functionality based on end-user feedback. As it progressed, the form transitioned to a portable, book-style shape, which end-users preferred for its ease of carrying, improved geometric integration into their current transport methods, and improved usability. An enhancement to SCRAMM’s functionality was the inclusion of three independent suction lines, which expands its operational capacity by enabling simultaneous management of multiple medical scenarios. The ability to perform different procedures at once offers versatility in the field, where time and efficiency are decisive. The final version of SCRAMM is illustrated in Fig. 2.

**Fig. 3:**
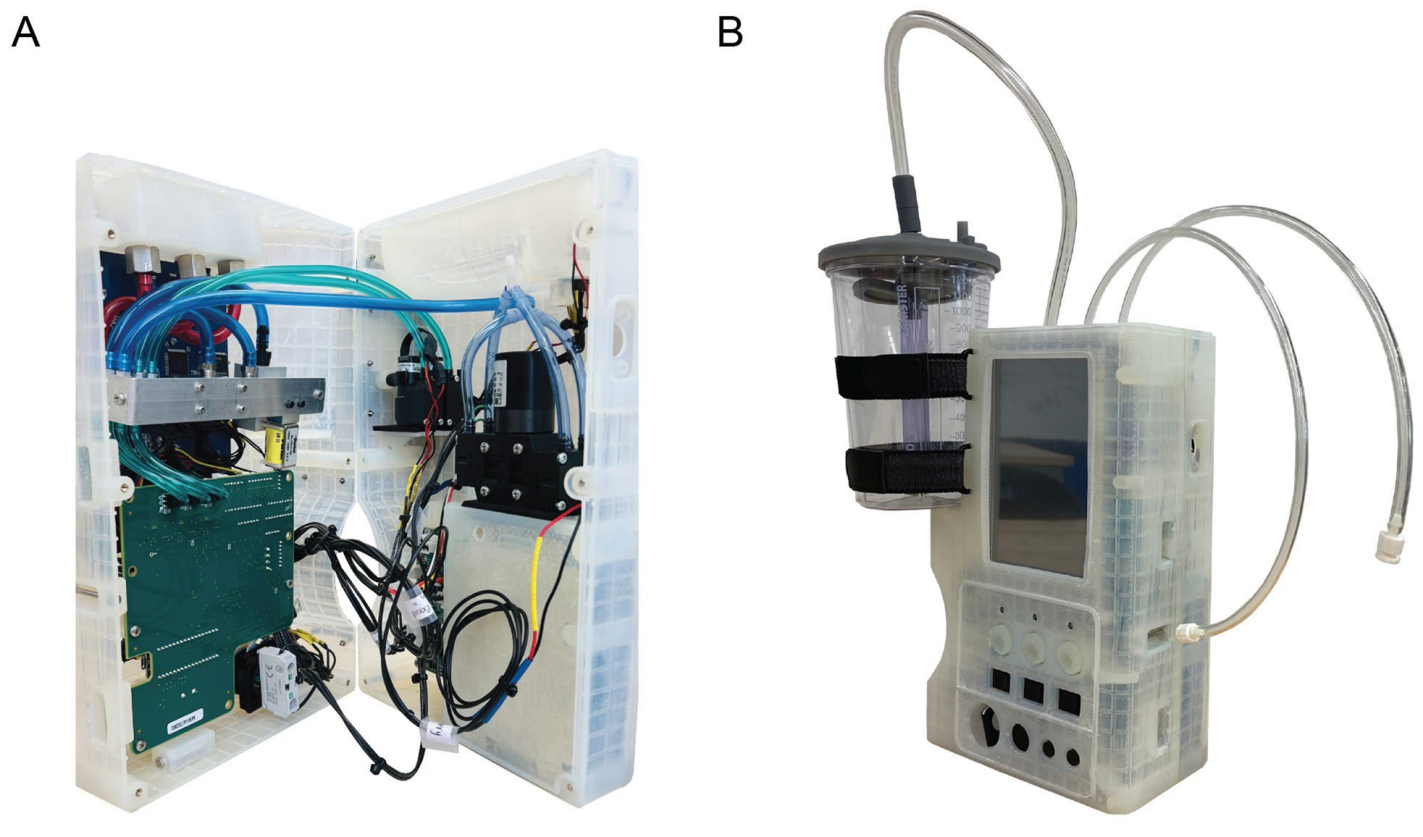
Prototyped version of SCRAMM. **A**: Internal view of the prototype with circuit boards, suction pumps and tube connections. **B**: Assembled prototype with suction line tube connections and canister as fluid collector

The prototype of the final version of the novel device is pictured in Fig. 3. The primary high-suction line, capable of delivering vacuum pressures up to 550 mmHg, is optimized for rapid airway clearance, oropharyngeal management, and surgical suction. The two secondary low-suction lines, with a maximum vacuum pressure of 150 mmHg, are designed for more delicate procedures like oral-nasal tracheal suction, dental suction, gastrointestinal abdominal drainage, wound suction, and chest tube suction. For enhanced usability, three distinct colors—yellow, blue, and red—differentiate the functions of the suction lines. Beyond the design elements related to its primary functionality, the electrical design of SCRAMM includes various controls and indicators, such as a power on/off switch, a pause/resume button, and a 7.0” screen for enhanced user interaction, facilitating the monitoring and adjustment of device settings.

### Phase 3: Testing

Following the prototype development, liquid flow rate tests were conducted and device weights were recorded to evaluate the performance of the three selected devices—SCRAMM, Zoll 330, and Impact 326M. This assessment aimed to determine how well each device balanced suction power and portability, leading to the proposal of a new performance-to-weight standard tailored to field use in military settings.

**Fig. 4:**
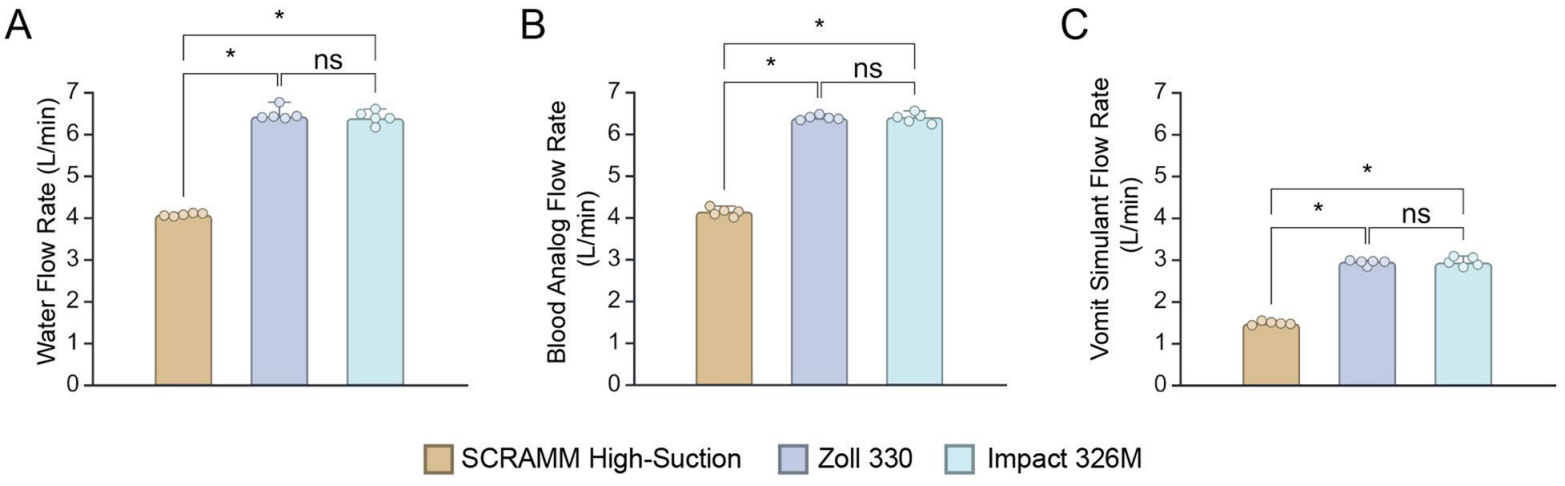
Results of the liquid flow rate tests for each solution. **A**–**C**: results of flow rate tests for the SCRAMM High-Suction line compared to Zoll 330 and Impact Ultra-lite 326M, across three different liquid simulants: (**A**) water, (**B**) blood analog, and (**C**) vomit simulant. Error bars represent 95% confidence interval, and asterisks represent statistical significance (* *p*$$$ < $$$ 0.05)

During the liquid flow rate test, the SCRAMM high-suction line achieved a flow rate of 4.07 L/min in the water tests, while the Zoll 330 and Impact 326M devices demonstrated higher flow rates of 6.48 L/min and 6.40 L/min, respectively. Statistical analysis using the Kruskal-Wallis test followed by Dunn’s multiple comparisons revealed statistically significant differences between SCRAMM and both competing devices (p $$$ < $$$ 0.05), as illustrated in Fig. 4A. Similarly in the blood analog tests, SCRAMM recorded a flow rate of 4.13 L/min whereas the Zoll 330 and Impact 326M achieved flow rates of 6.39 L/min and 6.38 L/min, respectively, with significant differences observed between SCRAMM and the other devices (p $$$ < $$$ 0.05), as shown in Fig. 4B. For the vomit simulant, SCRAMM exceeded the ISO 10079–1 standard of 1.2 L/min for viscous fluids, achieving a flow rate of 1.48 L/min—23% above the required threshold. In contrast, the Zoll 330 and Impact 326M surpassed the standard by 145%, with flow rates of 2.93 L/min and 2.95 L/min, respectively. Significant differences (p $$$ < $$$ 0.05) were again found between SCRAMM and the other two devices for vomit simulant flow rate, as depicted in Fig. 4C.

The weights of the SCRAMM, Zoll 330, and Impact 326M devices were measured at 3.4 kg, 4.8 kg, and 5.1 kg, respectively. Although the flow rate results demonstrated that the Zoll 330 and Impact 326M exceeded the ISO standard, they did so at the cost of added weight. Table 1 summarizes the testing results.

**Table 1:** Summarized weight and liquid flow rate performance of evaluated suction devices

Specification	SCRAMM	Zoll 330	Impact 326M
Weight (kg)	3.4	4.8	5.1
Water Liquid Flow Rate (L/min)	4.1	6.5	6.4
Blood Analog Liquid Flow Rate (L/min)	4.1	6.4	6.4
Vomit Simulant Liquid Flow Rate (L/min)	1.5	2.9	2.9

The flow rates reported for SCRAMM correspond to its high-suction line

Evaluating the flow rate and weight separately, however, does not fully reflect the operational needs in military contexts. While the flow rate determines the device’s ability to clear airway obstructions, its weight directly impacts its portability and usability in field conditions, as stated by end-users during the initial interviews [28]. As such, neither metric alone is sufficient to assess the real-world performance of the devices.

To better capture this balance between performance and portability, this study introduces a performance-to-weight metric. This metric quantifies the trade-off between suction performance and the ease of carrying and using the device in the field, factors deemed essential by end-users. Essentially, this metric identifies which device provides the best suction capability while remaining light enough to be practical in combat and emergency scenarios. A high-performing suction device that is too heavy may not be carried by medics, while a lightweight device that lacks sufficient suction may be ineffective.

**Fig. 5:**
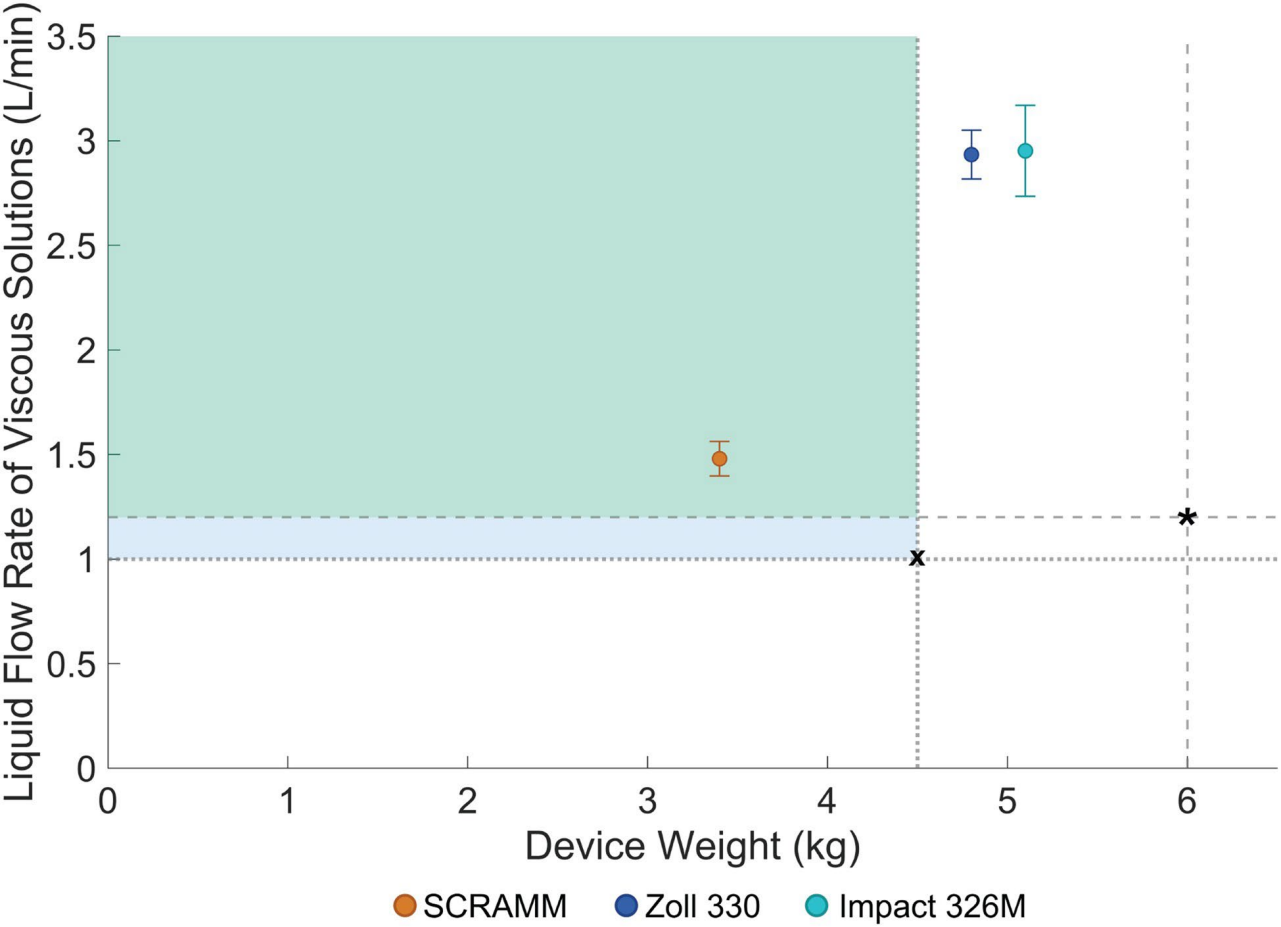
Proposed performance-to-weight standard for suction devices, comparing the SCRAMM, Zoll 330, and Impact 326M. Color dots represent mean liquid flow rates for viscous solutions, with error bars indicating 95% confidence intervals. The green shaded region denotes the proposed optimal standard, where devices meet both user-preferred weight limits and the required industry flow rate of 1.2 L/min for viscous solutions. The blue region highlights devices that, while lighter, fail to meet the required industry flow rate. The dotted lines intersecting at the ‘X’ symbol represent the maximum weight allowed by the end users (vertical) and the minimum flow rate allowed for end users (horizontal), while the dashed lines intersecting at the * symbol represent the maximum weight allowed by industry standards (vertical) and the minimum suction of viscous solutions allowed by industry standards (horizontal)

Figure 5 visually represents this concept. The dots constitute the mean values, and the error bars the 95% confidence intervals. The green shaded area indicates the new proposed performance-to-weight metric where devices achieve both the required flow rate for viscous solutions and maintain a weight that meets user expectations. The blue box represents areas outside the proposed criteria, particularly where devices do not meet the ISO-required flow rate for regulatory approval, even if they have a lighter weight.

Both the Zoll 330 and the Impact 326M meet the industry standards, which specify a maximum weight of 6 kg and a minimum liquid flow rate of 1.2 L/min for viscous solutions. However, these devices exceed the design requirement specified by end-users, which is under 4.5 kg. The SCRAMM prototype, at 3.4 kg, remains within the preferred weight range while also satisfying the required flow rate for industry standards; the SCRAMM prototype has the additional capabilities of two low-suction lines for simultaneous procedures.

## Discussion

The results of the liquid flow rate testing reveal a significant gap in the current industry standards for portable medical suction devices, particularly those used in military prehospital care. Effective suction devices are essential for airway management, as inadequate or delayed suctioning can lead to airway compromise—a leading cause of battlefield deaths and morbidity in trauma patients [1, 2, 9]. As evidenced in Figs. 4 and 5, although the Zoll 330 and Impact 326M meet the ISO 10079–1 standard for minimum liquid flow rates, this standard fails to address the critical need for portability—a key factor in field conditions.

The excessive weight of these market-leading devices, despite their adequate suction power, is a major drawback highlighted by combat medics and emergency responders [28]. This limitation directly impacts their usability in the field, where portability is as important as performance. The SCRAMM prototype was developed to balance performance and portability in response to the specific demands of military medical scenarios highlighted during end-user interviews. The device maintains a weight below the 4.5 kg threshold preferred by end-users while exceeding the industry standard for liquid flow rate. Additionally, the design includes three independent suction lines with variable pressures, providing versatility for different medical scenarios. However, while its lightweight design enhances portability, long-term durability under extreme conditions and cost-effectiveness compared to existing devices remain areas for further evaluation.

While the SCRAMM prototype addresses both performance and portability, current standards like ISO 10079–1 specify weight limits but fail to relate these directly to device performance. It could be argued that such standards should continue focusing on suction performance, thereby allowing the market to determine acceptable weight limits. However, this viewpoint assumes that the market efficiently identifies portable solutions; such an approach often proves unreliable. Products can claim to meet weight requirements without providing clear guidance on whether the weight compromises performance in real-world conditions. Therefore, we recommend that the ISO and other standard-setting bodies implement a performance-to-weight metric—ensuring that devices are not only powerful but also practical for use in the field.

Implementing such a metric not only addresses military needs but could also be extended to other emergency medical response fields. This metric could standardize the evaluation of portable suction devices in civilian emergency settings, such as paramedic units, disaster relief teams, and remote medical missions. Emergency responders often face transport constraints, yet current procurement decisions rely on manufacturer specifications without a clear standard to balance weight and performance. Integrating this metric into EMS procurement guidelines or FDA evaluations could help agencies select devices that optimize both mobility and suction efficiency—thus improving patient care in prehospital and disaster scenarios. Furthermore, this framework could be the groundwork for developing similar performance-to-weight metrics for other portable medical devices where both functionality and transportability are critical, such as ventilators, defibrillators, and diagnostic equipment used in emergency.

Limitations of this study include the controlled testing environments which, while designed to mimic field conditions, may not fully capture the unpredictable and varied circumstances of actual military operations. Conducting field tests in real operational settings would provide a more comprehensive evaluation of the device’s performance. Additionally, focusing primarily on technical and operational aspects leaves gaps in understanding the long-term durability and maintenance needs under continuous field use.

Furthermore, while the SCRAMM prototype demonstrates advantages in weight and versatility, potential drawbacks must also be considered. The durability of lightweight components under prolonged use in harsh environments remains to be fully assessed. Additionally, as a new design, the cost of production and deployment may be higher than existing market alternatives, which could influence large-scale adoption. These factors should be taken into account in future studies to ensure the device meets long-term operational needs.

Additionally, it is important to note that the SCRAMM device tested was still in the prototype stage. Transitioning from a prototype to a production-ready unit may introduce variations in material properties and overall performance. Therefore, it is essential to replicate all tests on the first production units to confirm these findings and ensure the device meets operational standards and user expectations in real-world conditions. Further, scaling SCRAMM for mass production presents additional challenges, including optimization of materials, production methods, and supply chain logistics. Ensuring consistent quality, meeting cost constraints, and navigating regulatory approvals such as FDA clearance will be key for real-world deployment. Ultimately, the ability to scale manufacturing efficiently may be a decisive factor in SCRAMM’s successful market adoption.

While this study primarily evaluated U.S. devices, expanding the scope to include European and global markets could provide insights for future iterations. Many European systems emphasize portability, modular canisters, and high-power suction—balancing performance with transportability. However, the systems are generally designed for short-term emergency use, with single-suction operation and limited battery life. In contrast, SCRAMM is designed for prolonged field care—offering a longer battery life and multiple independent suction lines to support simultaneous procedures. Future SCRAMM iterations could integrate aspects of European designs, such as suction components optimized for both power and weight efficiency, while maintaining its extended-use capabilities and multi-suction functionality to better meet the demands of diverse medical environments.

## Conclusion

This study highlights the need for revised standards in portable medical suction devices, especially in military prehospital care. While the Zoll 330 and Impact 326M meet current performance standards, their weight makes them impractical for field use. The SCRAMM prototype addresses this gap by offering a balanced combination of performance and portability—staying within the preferred weight range of under 4.5 kg while maintaining a liquid flow rate above the industry standard.

Given SCRAMM’s weight of 3.4 kg, users retain approximately 1.1 kg within the preferred 4.5 kg load limit. This remaining capacity may support mission-specific flexibility—whether to include suction accessories such as canisters or tubing, or to repurpose the allowance toward other essential medical equipment. Nonetheless, minimizing overall load remains a key priority, further underscoring the value of compact, multifunctional designs like SCRAMM.

To drive systemic improvements, we recommend that ISO and other standard-setting bodies revise medical suction device guidelines to better reflect the operational needs of field responders. This includes incorporating performance-to-weight considerations as well as factors like portability and multifunctionality. Ensuring that regulatory standards align with real-world usability needs would help guide the development of more practical and effective devices.

Further field testing and long-term durability evaluations will be essential for refining SCRAMM and validating its performance in real deployment conditions. Addressing these gaps will support the advancement and adoption of more efficient, field-ready medical suction solutions.

## Electronic supplementary material

Below is the link to the electronic supplementary material.

Supplementary Material 1: Title: Semi-Structured Interview Questions for NSF I-Corps program. Description of data: English version of interview questions used during NSF I-Corps Program.

Supplementary Material 2: Title: User Requirements and Product Specifications for Conceptual Design. Description of data: Two tables with the complete list of requirements and product specifications classified as physical performance and functional specifications

## Data Availability

The datasets used and/or analyzed during the current study are available from the corresponding author on reasonable request.

## Abbreviations

SCRAMM: Suction Combat Ready Advanced Multifunctional Machine
PFC: Prolonged Field Care
CBRNE: Chemical, biological, radiological, nuclear, and explosive
NSFI-Corps: National Science Foundation Innovation Corps
GUI: Graphical User Interface
FDA: Food and Drug Administration
3D: Three-dimensional
ISO: International Organization for Standardization
SLA: Stereolithography
