# Supplementary material for: Enhancing military airway suction devices with a focus on performance and portability
Source: BMC Emerg Med. 2025 Jul 16;25:128. doi: 10.1186/s12873-025-01262-4 (PMC12269139; doi:10.1186/s12873-025-01262-4)
Supplement: Supplementary file 1 — Supplementary Material 1: Title: Semi-Structured Interview Questions for NSF I-Corps program. Description of data: English version of interview questions used during NSF I-Corps Program. [file 12873_2025_1262_MOESM1_ESM.pdf]

Additional File 1

Semi-Structured Interview Questions for NSF I-Corps program

As part of the NSF I-Corps program, the following semi-structured interview questions were posed to end-users to assess needs related to suction device use. While these eight core questions were asked to all participants, additional questions may have been introduced based on the flow of conversation:

1. Are you a medical professional that has had to triage patients?
2. Were you in-hospital or out-of-hospital?
3. Talk to me about the need for suction in the field.
4. Do you use suction for airway management?
5. What equipment did you use outside of the hospital for airway management?
6. What were some of the problems you had in the field?
7. How much does equipment cost?
8. Is suction a procedure that is billable? Or are those costs passed on under another service/procedure like intubation?
