# Supplementary material for: Enhancing military airway suction devices with a focus on performance and portability
Source: BMC Emerg Med. 2025 Jul 16;25:128. doi: 10.1186/s12873-025-01262-4 (PMC12269139; doi:10.1186/s12873-025-01262-4)
Supplement: Supplementary file 2 — Supplementary Material 2: Title: User Requirements and Product Specifications for Conceptual Design. Description of data: Two tables with the complete list of requirements and product specifications classified as physical performance and functional specifications [file 12873_2025_1262_MOESM2_ESM.pdf]

Additional File 2

User Requirements and Product Specifications for Conceptual Design

Table 1 User requirements list obtained from NSF's I-Corps program interviews

User Requirement	Description
Portability	Device should be portable, as both a compact design and low carry weight
Efficiency	Device should provide fast and effective suction
Operational Flexibility	Device should provide suction in any orientation
Volume Capacity	Device should evacuate a volume of at least 1 L
Obstruction Management	Device should overcome large particulate obstruction
Power Source Versatility	Device should be battery and wall operated
Infection Control	Device should not transmit any kind of virus from patient to user
Sterilization Compatibility	Device should be sterilizable

Table 2 Product specifications and design requirements categorized between physical, performance, and functional specifications

Physical Specifications	
Specifications	Design Requirements
Overall Dimensions without Canister	$\leq$ 21 x 32 x 12 cm
Volume	$\leq$ 8064 cm^3
Weight	$\leq$ 4.5 kg
Canister Capacity	$\geq$ 1000 mL
**Performance Specifications**	
Specifications	Design Requirements
Liquid Flow Rate	$\geq$ 1 L/min
Orientation Independency	Canister design to be orientation independent
Vacuum Pressure at Suction Tip	Range: 10 – 550 mmHg
Device Operation Time	Minimum operational time of 8.5 hours under maximum load; targets 2 hours continuous operation with an additional 6 hours as a safety factor based on end-user feedback and competitor analysis
Device Operation Type	Battery-operated with a voltage range of 12 – 24 VDC<br>Power grid operation with a voltage range of 120 - 240 VAC
Operational Temperature Range	Device shall operate within the temperature specification mentioned in military standards for medical devices (0 – 60 °C)
Device Robustness	Drop Test: Device must withstand a drop from 1 m onto a hard surface (e.g., concrete)<br>Vibration Test: Device must withstand vibration at a frequency of 1000 Hz for 20 minutes
Maximum Noise Level for Overall Device	$\leq$69 dB
Suction Tube Length	1 m
Suction Tube Diameter	Inner Diameter (ID) 12 mm
Viscosity	Device must perform suction of liquids with viscosities up to 25 cP
**Functional Specifications**	
Specifications	Design Requirements
Indicator for suction	Indicator light activates when suction is operational
Viral filtration to protect caregivers	Device must incorporate hydrophobic viral filters to prevent aerosolized pathogen spread
Sterilizability	Device components shall be disposable and capable of being sterilized
Low battery indication	Battery life shall be monitored via an external display
Variable pressures available at need	Graphical user interface shall allow access to different suction pressures and operational modes as required (e.g., intermittent suction)
Multiple Suction Capability	Device must independently provide suction through two or more hoses at variable pressures
